# RETRACTION: Downregulation of HBx Restrains Proliferation, Migration, and Invasion of HepG2 Cells

**DOI:** 10.1155/ancp/9835195

**Published:** 2025-11-19

**Authors:** Analytical Cellular Pathology

RETRACTION: C. Huang, W. Liu, X. Zhao, L. Zhao, and F. Wang, “Downregulation of HBx Restrains Proliferation, Migration, and Invasion of HepG2 Cells,” *Analytical Cellular Pathology* 2021 (2021): 6615979, https://doi.org/10.1155/2021/6615979.

The above article, published online on 15 May 2021 in Wiley Online Library (wileyonlinelibrary.com), has been retracted by agreement between the journal Editor‐in‐Chief, Dimitrios Karamichos; and John Wiley & Sons Ltd.

The retraction has been agreed following an investigation of the concerns raised by *Hoya camphorifolia* on PubPeer [1], which identified an instance of figure duplication.

Specifically, the HepG2 colonies in the "NC" and "Si‐HBX+ XB130" cell plates depicted in Figure 4c share unexpected similarities with the "Control" and "MWNT" cell plates in Figure 2b of [2] when rotated by 90 degrees.

As a result of the investigation, the data and conclusions of this article are considered unreliable.

The authors have been informed of this retraction but did not provide a response.
